# Endovascular thrombectomy in patients with anterior circulation stroke: an emulated real-world comparison

**DOI:** 10.1186/s42466-024-00331-6

**Published:** 2024-07-25

**Authors:** Jochen A. Sembill, Maximilian I. Sprügel, David Haupenthal, Svenja Kremer, Michael Knott, Iris Mühlen, Bernd Kallmünzer, Joji B. Kuramatsu

**Affiliations:** 1 Department of Neurology, Friedrich-Alexander-Universität Erlangen-Nürnberg (FAU), Schwabachanlage 6, 91054 Erlangen, Germany; 2 Department of Neuroradiology, Friedrich-Alexander-Universität Erlangen-Nürnberg (FAU), Schwabachanlage 6, 91054 Erlangen, Germany

**Keywords:** Stroke, Thrombectomy, Treatment

## Abstract

**Background:**

Endovascular thrombectomy (EVT) has been proven effective in anterior circulation stroke due to large vessel occlusion (LVO). However, translation from randomized clinical trials (RCTs) with highly selected patients to real-world requires confirmation, particularly to identify associations outside of strict selection criteria.

**Aims:**

This study aims to compare functional outcomes after EVT in real-world with those reported in RCTs, and to identify associations with functional outcome after EVT outside RCT-criteria.

**Methods:**

This study analyzed longitudinal German real-world data from the Stroke Research Consortium in Northern Bavaria (STAMINA) cohort from January, 2015 to June, 2019. We conducted a trial emulation, comparing patients with anterior circulation stroke and LVO meeting selection criteria for RCTs investigating EVT (1) predominantly within 6 hours with those from HERMES meta-analysis, and (2) within 6-24 hours with those from AURORA meta-analysis. We (3) analyzed treatment effects of EVT and association with functional outcome in patients treated outside RCT criteria.

**Results:**

Of 598 patients, 281 (47.0%) met RCT-criteria for treatment within 6 hours (hereinafter STAMINA-HERMES), 74 (12.4%) met RCT-criteria for treatment within 6–24 hours (STAMINA-AURORA), and 277 (46.3%) patients received EVT outside RCT-criteria. We observed no difference in rates of functional independence or mortality, comparing STAMINA-HERMES with HERMES meta-analysis (mRS 0-1: *n*=120/281 [43%] vs. 291/633 [46%], *p*=0.36; mortality: *n*=34/281 [12%] vs. 97/633 [15%], *p*=0.20), and STAMINA-AURORA with AURORA meta-analysis (mRS 0-1: *n*=26/74 [35%] vs. 122/266 [46%], *p*=0.10, mortality: *n*=10/74 [14%] vs. 45/266 [17%], *p*=0.48). Patients treated outside RCT-criteria had worse outcome (mRS 0-1: *n*=38/277 [14%], mortality: *n*=90/277 [32%], both *p*<0.001); possibly driven by pre-existing functional dependence (*n*=172/277 [62%]). Compared to matched controls, EVT outside of RCT-criteria was associated with lower mortality (absolute treatment effect: -14%, 95% Confidence Interval [CI] -23 to -5, *p*<0.01), but not with recovery to functional independence or premorbid functional status (treatment effect: 4%, CI -4 to 11, *p*=0.34), which was associated with lower NIHSS (Odds ratio [OR] 0.86, CI 0.80-0.92, *p*<0.001) and age (OR 0.95, CI 0.93-0.98, *p*=0.002).

**Conclusions:**

Translation of EVT outcomes reported in RCTs into real-world is possible, however, almost half of patients did not meet trial criteria. Identification of patients who functionally benefit from frequently performed EVT outside RCT-criteria requires further investigation.

**Trial registration:**

Clinicaltrials.gov, NCT04357899.

**Supplementary Information:**

The online version contains supplementary material available at 10.1186/s42466-024-00331-6.

## Introduction

Endovascular thrombectomy (EVT) is of high efficacy and safety for treatment of large vessel occlusion (LVO) in patients with acute anterior circulation stroke [1]. This was demonstrated for patients treated predominantly within 6 hours of time last seen well by five large randomized controlled trials (RCT) published in 2015 (MR CLEAN [2], ESCAPE [3], REVASCAT [4], SWIFT PRIME [5], and EXTEND IA [6]) and first meta-analyzed by the HERMES [1] collaboration (functional independency, Intervention: 46% vs. Control: 27%). Since 2018, results from subsequent trials (DAWN [7], DEFUSE 3 [8], RESILIENT [9], POSITIVE [10]) documented benefit of endovascular thrombectomy in patients with salvageable brain tissue across within a time window of 6-24 hours, which was investigated by the recent AURORA [11] meta-analysis (functional independency, Intervention: 46% vs. Control: 19%). However, translating data from clinical trials with highly selected patients into the real world is complex [12]. Observational data suggest lower rates of favorable outcome in clinical practice (26% [13] to 37% [14]), but many patients were treated outside RCT inclusion criteria [15]. Only limited data exist regarding the outcome of patients meeting inclusion criteria in the real world compared with reported outcome of patients treated in RCTs [15, 16]. Prior studies investigated only selected trials or few patients fulfilling extended time window inclusion criteria with limited perfusion imaging data [15, 16]. Furthermore, there is limited information on which patients also benefit from endovascular therapy despite not meeting RCT selection criteria.

## Aims

The present study analyzed longitudinal real-world data from patients with acute anterior circulation stroke treated with EVT within the framework of a large stroke telemedicine network. We conducted a real-world trial emulation, aiming to compare (1) functional outcome of patients meeting inclusion criteria for trials investigating EVT within the 6 hour time window with the results of the HERMES meta-analysis, and (2) functional outcome of patients meeting inclusion criteria for trials investigating EVT within the 6-24 hour time window with the results of the AURORA meta-analysis. In addition, we investigated (3) treatment effects of EVT and association with functional outcome in patients treated outside clinical trial criteria.

## Methods

### Study design & participants

The longitudinal STAMINA (Stroke Research Consortium in Northern Bavaria, www.clinicaltrials.gov; NCT04357899) study included consecutive patients with ischemic stroke admitted to the University Hospital Erlangen, Germany, as the coordination center of a large telemedicine network between January 01, 2006 and June 30, 2019 [17]. The present analysis investigated patients with acute anterior ischemic stroke due to LVO treated with EVT ± thrombolysis between January 2015 and June 2019 as EVT was routinely performed during this time period [17]. The local ethics committee of the Friedrich-Alexander-University Erlangen-Nuremberg, Germany reviewed and approved the study (Registration No 62_21B). Patients or their legal representatives provided informed consent unless centrally waived by the review board [17].

### Definitions and data acquisition

We obtained data on demographics, comorbidities, prestroke functional status, hospital admission status, intra-hospital clinical and radiological parameters, as previously described [17–19]. Follow-up data were obtained by trained raters at 90 days by telephone interview, outpatient visits, or medical reports [17]. Outcome was assessed using the modified Rankin Scale (mRS), ranging from normal (0) to severe disability (5) and death (6) (1). We applied the trials’ exclusion and inclusion criteria of MR CLEAN (2), ESCAPE (3), REVASCAT (4), SWIFT PRIME (5), and EXTEND IA (6) as well as of DAWN (7), DEFUSE 3 (8), RESILIENT (9), POSITIVE (10) to our cohort to identify patients fulfilling criteria for participation into an emulated real world cohort. For an overview of main trial criteria see Supplemental Table 1.

### Outcomes

The primary outcome was the proportion of patients with functional independence at 90 days (mRS 0–2). Secondary outcomes comprised (i) mortality at 90 days, and (ii) a composite endpoint of neurological recovery to functional independence or to pre-morbid functional status at 90 days among patients treated outside trial criteria.

### Statistical analyses

We performed statistical analyses using the Stata software (StataCorp. 2021. Stata Statistical Software: Release 17. College Station, TX: StataCorp LLC) and IBM SPSS Statistics (IBM Corp. Released 2016. IBM SPSS Statistics for Windows, Version 24.0. Armonk, NY: IBM Corp.). We applied two-sided statistical tests with a significance level of 0.05. We report categorical variables as total numbers and percentages, evaluated by the χ^2^ test or Fisher exact test, and ordinal and continuous variables as medians and interquartile ranges (IQRs), evaluated by the Mann-Whitney test. We compared characteristics and outcomes of patients fulfilling all inclusion criteria and no exclusion criteria of at least one of MR CLEAN, ESCAPE, REVASCAT, SWIFT PRIME, or EXTEND IA and treatment predominantly within 6 hours with patients included into the HERMES meta-analysis. In the same way, we compared patients fulfilling all inclusion criteria and no exclusion criteria of at least one of DAWN, DEFUSE 3, RESILIENT, or POSITIVE and treatment within the 6-24 h time window with patients included into the AURORA meta-analysis. As enrolment in ESCAPE and REVASCAT was also possible beyond 6 hours, patients could fall into the time category with EVT predominantly within 6 hours as well as 6-24 hours [1, 11]. Consequently, some patients were able to be included in both emulation cohorts for comparison with patients included in both meta-analyses. We further compared outcomes of patients within the STAMINA cohort received EVT inside vs. outside trial criteria. Among patients treated with EVT outside mentioned trial criteria, we recorded main reasons for exclusion and conducted stepwise multivariable regression modeling to evaluate exclusion criteria for association with functional outcome. We investigated independent associations with a composite endpoint of neurological recovery to functional independence or to pre-morbid functional status at 90 days among patients treated outside trial criteria. To limit confounding, we conducted propensity score matching of patients treated with EVT outside clinical trial criteria and of patients with anterior ischemic stroke due to large vessel occlusion not treated with EVT to calculate the adjusted absolute treatment effects of EVT by augmented inverse probability weighting [20].

## Results

### Patient characteristics

**Fig. 1:**
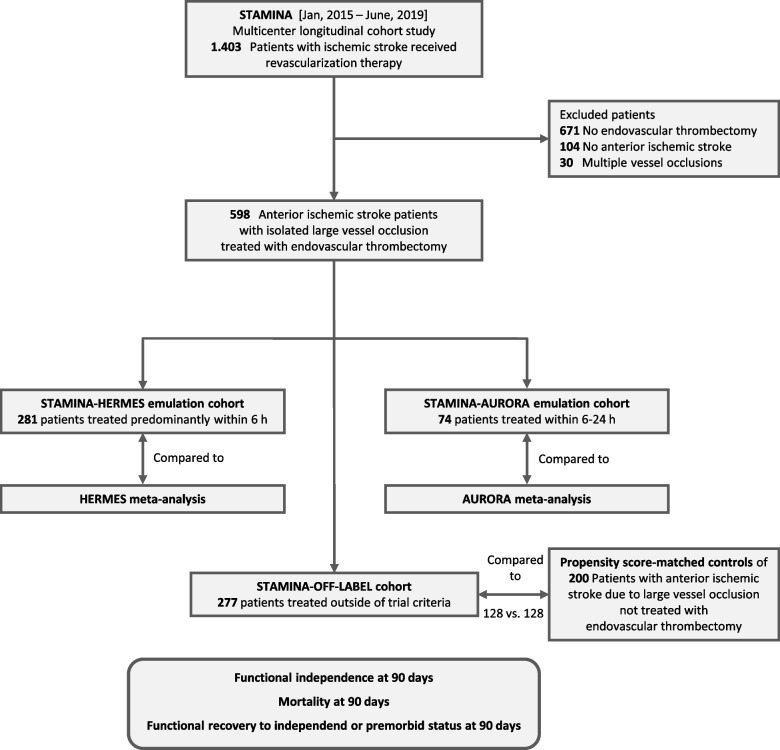
Study flow chart. Shown is the flow chart of patients included into the Stroke Research Consortium in Northern Bavaria (STAMINA) cohort study. After exclusion of patients without endovascular thrombectomy, anterior ischemic stroke, or multiple vessel occlusion, data from 598 patients with anterior ischemic stroke and isolated large vessel occlusion were analyzed. Depending on whether the inclusion criteria of the study were met, patients were assigned to the STAMINA-HERMES or the STAMINA-AURORA emulation cohort (or both, *n*=34) or to the STAMINA-OFF-LABEL cohort of patients who received thrombectomy outside the trial inclusion criteria. Outcomes were compared with those of the HERMES meta-analysis, the AURORA meta-analysis, and the matched controls, respectively

Of 1,403 acute ischemic stroke patients recruited in the STAMINA cohort study, there were 598 patients with isolated acute LVO in the anterior circulation treated with EVT with or without thrombolysis, see Fig. 1 for study flow chart. 281 out of 598 (47.0%) patients were treated predominantly within 6 h of time last seen well and in-line with trial criteria of at least one of the trials MR CLEAN, ESCAPE, REVASCAT, SWIFT PRIME, or EXTEND IA (hereinafter referred to as the STAMINA-HERMES emulation cohort), and 74 out of 598 (12.4%) patients were treated within the 6–24 h time window and in-line with trial criteria of at least one of the trials DAWN, DEFUSE 3, RESILIENT, or POSITIVE (hereinafter referred to as the STAMINA-AURORA emulation cohort). As 34 patients met the criteria of RCTs included in both the HERMES cohort and the AURORA cohort, they were analyzed in both cohorts. The remaining 277 (46.3%) patients received EVT outside mentioned trial criteria (hereinafter referred to as the STAMINA-OFF-LABEL cohort).

Table 1 compares patients from the STAMINA-HERMES emulation cohort with those from the HERMES meta-analysis. Real-world patients were older [73 years (58-79) vs. 68 years (57–77)] and had more comorbidities [hypertension, *n*=211/281 (75%) vs. *n*=352/634 (56%); diabetes mellitus, *n*=76/211 (27%) vs. *n*=82/634 (13%); atrial fibrillation, *n*=123/281 (44%) vs. *n*=209/634 (33%)], more frequent occlusion of the internal carotid artery [*n*=98/211 (35%) vs. *n*=133/634 (21%)] or the M2 segment of middle cerebral artery [*n*=41/211 (15%) vs. *n*=51/634 (8%)], and received intravenous alteplase as frequently but later than patients in the HERMES cohort [intravenous alteplase within 180 min, *n*=152/281 (54%) vs. *n*=442/634 (70%)]

**Table 1:** Characteristics of patients from the STAMINA-HERMES emulation cohort compared to those of the HERMES meta-analysis

**Parameters**	STAMINA-HERMES emulation cohort (*n*=281)	HERMES meta-analysis (*n*=634)
Demographics		
Age, median (IQR), years	73 (58-79)	68 (57–77)
Female Sex, No.(%)	143 (51)	304 (48)
Prior medical history		
Hypertension, No.(%)	211 (75)	352 (56)
Diabetes mellitus, No.(%)	76 (27)	82 (13)
Atrial fibrillation, No.(%)	123 (44)	209 (33)
Smoking (recent or current), No.(%)	71 (25)	194 (31)
Clinical characteristics		
Baseline NIHSS Score^a^, median (IQR)	15 (11-18)	17 (14–20)
Imaging characteristics		
Baseline ASPECT Score^b^, median (IQR)	9 (7-10)	9 (7–10)
Intracranial occlusion location		
Internal carotid artery, No.(%)	98 (35)	133 (21)
Middle cerebral artery, M1 segment, No.(%)	141 (50)	439 (69)
Middle cerebral artery, M2 segment, No.(%)	41 (15)	51 (8)
Anterior cerebral artery, No.(%)	1 (0)	11 (2)
Treatment		
Treatment with intravenous alteplase, No.(%)	223 (79)	526 (83)
Treatment with intravenous alteplase within 180 min, No.(%)	152 (54)	442 (70)
Process times		
Onset to intravenous alteplase, (min)	94 (75-129)	100 (75–133)
Onset to reperfusion, (min)	310 (221-403)	285 (210–362)

*Abbreviations*: *IQR* Interquartile range (25th-75th percentile)

^a^NIH Stroke Scale, range 0-42, level of neurological impairment after stroke

^b^Alberta Stroke Program Early CT Score, range 0-10, assessment of early ischemic changes in acute stroke

Table 2 compares patients from the STAMINA-AURORA emulation cohort with those from the AURORA meta-analysis. Real-world patients were older [74 years (60-79) vs. 68 years (±14)], had more frequent occlusion of the internal carotid artery [*n*=34/74 (46%) vs. *n*=66/266 (25%)] than of the middle cerebral artery [*n*=39/74 (53%) vs. *n*=93/266 (73%)], and received intravenous alteplase more frequent than patients in the AURORA cohort [*n*=41/74 (55%) vs. *n*=28/266 (11%)].

**Table 2:** Characteristics of patients from the STAMINA-AURORA emulation cohort compared to those of the AURORA meta-analysis

**Parameters**	STAMINA-AURORA emulation cohort (*n*=74)	AURORA meta-analysis (*n*=266)
Demographics		
Age, median (IQR) / mean (SD), years	74 (60-79)	68 (±14)
Female Sex, No.(%)	38 (51)	146 (55)
Prior medical history		
Hypertension, No.(%)	62 (84)	195 (74)
Diabetes mellitus, No.(%)	18 (24)	68 (26)
Atrial fibrillation, No.(%)	34 (46)	96 (39)
Hyperlipidaemia, No.(%)	38 (51)	138 (53)
Clinical characteristics		
Baseline NIHSS Score^a^, median (IQR)	14 (11-18)	16 (13–20)
Imaging characteristics		
Baseline ASPECT Score^b^, median (IQR)	8 (6-9)	8 (7–9)
Intracranial occlusion location		
Internal carotid artery, No.(%)	34 (46)	66 (25)
Middle cerebral artery, M1 segment, No.(%)	39 (53)	193 (73)
Middle cerebral artery, M2 segment, No.(%)	1 (1)	7 (3)
Treatment		
Treatment with intravenous alteplase, No.(%)	41 (55)	28 (11)
Process times		
Onset to puncture, (min)	590 (412-881)	662 (508–809)
Onset to reperfusion, (min)	700 (500-925)	719 (572–911)

*Abbreviations*: *IQR* Interquartile range (25th-75th percentile)

^a^NIH Stroke Scale, range 0-42, level of neurological impairment after stroke

^b^Alberta Stroke Program Early CT Score, range 0-10, assessment of early ischemic changes in acute stroke

Supplemental Table 2 compares patients included either into the STAMINA-HERMES or the STAMINA-AURORA emulation cohort (*n*=321) with patients received EVT outside trial selection criteria, i.e. the STAMINA-OFF-LABEL cohort. Patients from the STAMINA-OFF-LABEL cohort were older [78 years (67–84) vs. 73 years (59-79), *p*<0.001] and more frequent female [*n*=169/277 (61%) vs. *n*=166/321 (52%), *p*=0.02], had higher premorbid functional dependence [premorbid mRS, 2 (0-3) vs. 0 (0-1), *p*<0.001], lower initial mismatch volume in perfusion imaging [94 mL (40-140) vs. 105 mL (71-153), *p*=0.02], more frequent peripheral occlusion of the middle cerebral artery [M2 segment, *n*=57/277 (21%) vs. *n*=42/321 (13%), *p*=0.01], less frequent and later treatment with intravenous alteplase [*n*=161/277 (58%) vs. *n*=245/321 (76%), *p*<0.001; time from onset to intravenous alteplase, 115 min (90–173) vs. 95 min (75-130), *p*=0.002], and longer time from onset to reperfusion [465 min (314–924) vs. 325 min (238-464), *p*<0.001] with lower reperfusion rates [TICI scale ≥2b, *n*=230/277 (83%) vs. *n*=296/321 (92%), *p*<0.001].

### Outcomes

**Fig. 2:**
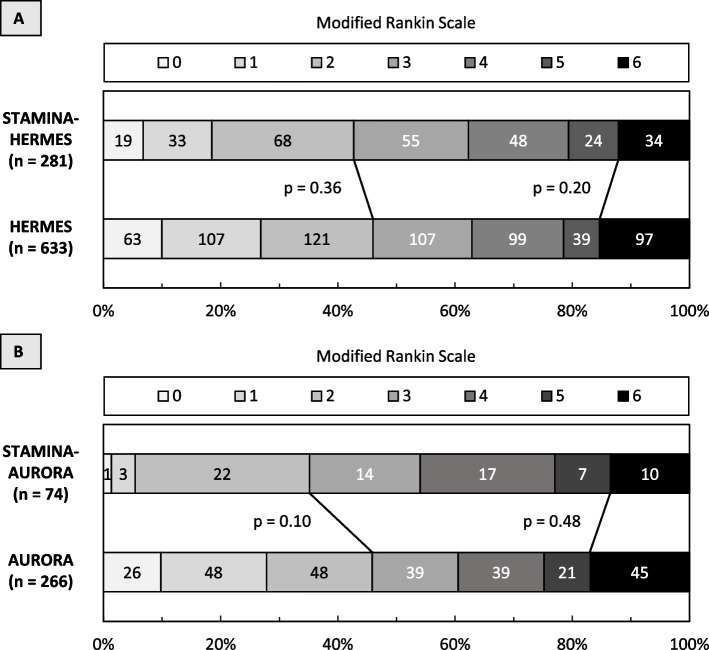
Functional outcome in the STAMINA emulation cohorts vs. in meta-analyses. Shown is the distribution of scores on the modified Rankin scale after 90 days (**A**) in patients treated predominantly within 6 hours from the STAMINA-HERMES emulation cohort vs. patients from the HERMES meta-analysis, and (**B**) in patients treated predominantly within 6-24 hours from the STAMINA-AURORA emulation cohort vs. patients from the AURORA meta-analysis. The Scores range from 0 to 6, with 0 indicating no symptoms, 1 no clinically significant disability, 2 slight disability, 3 moderate disability, 4 moderately severe disability, 5 severe disability, and 6 death. Scores from 0 to 2 are considered functional independence. There were no significant differences in the proportions of functional independence or mortality between the compared groups

Comparing patients from the STAMINA-HERMES emulation cohort with those from the HERMES meta-analysis, we observed no difference in the proportion of patients with functional independence at 90 days (STAMINA: *n*=120/281 [43%] vs. HERMES: 291/633 [46%], *p*=0.36) or in mortality (STAMINA: *n*=34/281 [12%] vs. HERMES: 97/633 [15%], *p*=0.20), see Fig. 2A.

Comparing patients from the STAMINA-AURORA emulation cohort with those from the AURORA meta-analysis, we observed no significant difference in the proportion of patients with functional independence at 90 days (STAMINA: *n*=26/74 [35%] vs. AURORA: 122/266 [46%], *p*=0.10) or in mortality (STAMINA: *n*=10/74 [14%] vs. AURORA: 45/266 [17%], *p*=0.48), see Fig. 2B.

Comparing patients included either into the STAMINA-HERMES or the STAMINA-AURORA emulation cohort with patients from the STAMINA-OFF-LABEL cohort, we observed fewer patients achieving functional independence after 90 days and higher mortality rates among patients not in line with trial inclusion criteria (mRS 0-2, emulation cohort: *n*=131/321 [41%] vs. STAMINA-OFF-LABEL: *n*=38/277 [14%], *p*<0.001; mortality, emulation cohort: *n*=42/321 [13%] vs. STAMINA-OFF-LABEL: *n*=90/277 [32%], *p*<0.001), see Supplemental Figure 1.

### Main exclusion reasons for trial participation

The most common exclusion reason for potential trial participation reason was a prestroke functional dependence, i.e. mainly mRS ≥2, in 172 of 277 (62%) patients, followed by large infarct core respectively low ASPECTS (*n*=44/277 [16%]), unknown onset or onset >16 respectively >24 hours (*n*=44/277 [16%]), peripheral artery occlusion (*n*=26/277 [9%]), too-low NIHSS score (*n*=21/277 [8%]) (multiple classification possible), among other less common reason (including lack of perfusion imaging if required, life-limiting disease, severe renal failure, sensitivity to contrast agents, or hemorrhagic diathesis). To assess the relevance of exclusion criteria for functional outcome, we performed stepwise multivariable modeling showing negative association of pre-stroke functional dependence with subsequent functional independence (Odds ratio [OR] 0.07, 95% Confidence Interval [CI] 0.02-0.22, *p*<0.001), and positive association with peripheral artery occlusion (OR 2.74, CI 1.11-6.78, *p*=0.03) and too low NIHSS score (OR 7.01, CI 2.39-20.58, *p*<0.001).

### Association with functional outcome among patients from the STAMINA-OFF-LABEL cohort

Due to high rate of premorbid functional impairment, i.e. 62% of patients, we assessed a composite endpoint of recovery to functional independence or premorbid functional status to identify associations with favorable functional outcome after EVT in the STAMINA-OFF-LABEL cohort. Patients who achieved functional recovery (*n*=62/277, 22%), compared to those who did not (*n*=215/277, 78%), were younger [69 (55-79) vs. 80 (72–85), *p*<0.001], were less often female [*n*=31/62 (50%) vs. *n*=138/215 (64%), *p*=0.04], had lower NIHSS [10 (5–16) vs. 17 (13-21), *p*<0.001], had less frequent comorbidities [arterial hypertension: *n*=44/62 (71%) vs. *n*=184/215 (86%), *p*=0.01; atrial fibrillation [*n*=20/62 (32%) vs. *n*=109/215 (51%), *p*=0.01] and oral anticoagulation [*n*=6/62 (10%) vs. *n*=46/215 (21%), *p*=0.04], and had a higher ASPECT Scorer [10 (8-10) vs. 9 (7-10), *p*=0.04] respectively smaller ischemic core lesion volume [0 mL (0-19) vs. 8 mL (0-38), *p*=0.04], see Supplemental Table 3. Multivariable analysis showed independent association of functional recovery only with lower NIHSS (OR 0.86, CI 0.80-0.92, *p*<0.001) and younger age (OR 0.95, CI 0.93-0.98, *p*=0.002).

### Treatment effects of EVT in the STAMINA-OFF-LABEL cohort

**Fig. 3:**
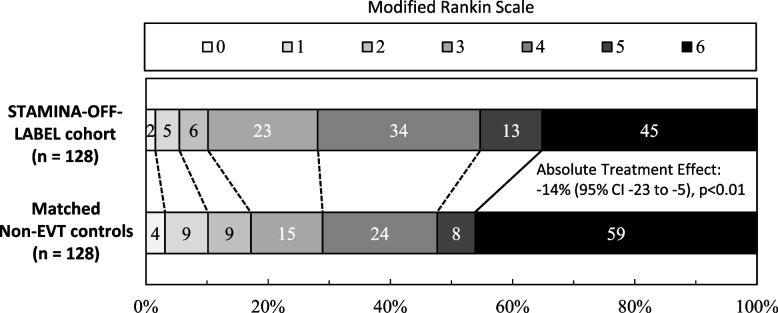
Functional outcome of patients from the STAMINA-OFF-LABEL cohort compared to propensity score-matched controls without endovascular thrombectomy. Shown is the distribution of scores on the modified Rankin scale (mRS) at 90 days in patients from the STAMINA-OFF-LABEL cohort treated with anterior ischemic stroke due to large vessel occlusion treated with endovascular thrombectomy (EVT) outside clinical trial criteria, compared to propensity score-matched Non-EVT controls, i.e. patients with anterior ischemic stroke due to large vessel occlusion not treated with EVT. The Scores range from 0 to 6, with 0 indicating no symptoms, 1 no clinically significant disability, 2 slight disability, 3 moderate disability, 4 moderately severe disability, 5 severe disability, and 6 death. We conducted adjusted Treatment‐effects estimation of EVT for mortality (i.e. mRS 6, solid line) as well as recovery to functional independence (i.e. mRS 0-2) or premorbid functional status [dashed lines; Absolute Treatment Effect: 4% (95% CI -4 to 11), *p*=0.34]. Abbreviations: CI, Confidence Interval

To investigate treatment effects of EVT in the STAMINA-OFF-LABEL cohort, we compared patients from the STAMINA-OFF-LABEL cohort with controls with anterior circulation stroke due to LVO not treated with EVT (Supplemental Table 4A). We controlled for unbalanced patient and treatment characteristics, i.e. age, pre-mRS, ASPECT Score, ischemic core lesion and mismatch volume, and treatment with intravenous alteplase, using propensity score matching to obtain evenly balanced cohorts of 256 patients (Supplemental Table 4B). EVT was associated with lower mortality at 90 days [absolute treatment effect: -14% (95% confidence interval: -23 to -5), *p*<0.01], but with no association with the composite endpoint of recovery to functional independence or premorbid functional status [absolute treatment effect: 4% (95% confidence interval: -4 to 11), *p*=0.34], see Fig. 3.

## Discussion

The present emulated RCT to real-world comparison demonstrated that the translation of outcome data from rigorously selected patients with anterior ischemic stroke treated with EVT in RCTs to the real world is possible; both in the 0-6 hour time window and in the 6-24 hour time window. However, almost half of patients did not meet trial criteria, mainly because of preexisting functional dependence. Comparing these patients with propensity matched controls, EVT was only associated with reduced mortality without being associated with improved functional outcome. Lower age and lower NIHSS was associated with functional recovery to independent or pre-morbid status at 90 days after EVT outside of clinical trial criteria.

The comparable results after EVT in RCTs vs. in the real world are encouraging, as patients of the STAMINA emulation cohorts are characterized by older age and more frequent comorbidities despite strict application of the appropriate RCT inclusion criteria. The patients eligible for participation in the evaluated RCTs represented a strictly selected subgroup, so verification of outcomes in routine clinical practice is important [21]. Thus, the present data support the now widespread use of EVT, as suggested by prior registries and observational studies [15, 16, 22]. However, to our knowledge, this is the first emulation study strictly considering inclusion criteria of the nine major RCTs for EVT in anterior circulation ischemic stroke. Our potential cumulative RCT participation rate of 54% is therefore higher than rates in a previous analysis of a German multicenter prospective registry reporting only potential participation in five selected trials (MR CLEAN, 35%; ESCAPE, 14%; SWIFT‐PRIME, 9%; DAWN, 4%; and DEFUSE‐3, 3%) [15]. The higher rate reported here is probably due to the larger number of RCTs considered simultaneously and the more comprehensive access to detailed perfusion data in this study compared to this registry [15]. A similar parameter – the proportion of EVT in accordance with the American Heart Association/American Stroke Association (AHA/ASA) recommendations at the time of the study (EVT ≤6 hours of symptom onset, intravenous tissue plasminogen activator (if eligible), NIHSS ≥6, anterior circulation occlusion only – was analyzed by the TREVO Stent-Retriever Acute Stroke (TRACK) multicenter registry. The results indicated that only 26% of patients were treated in accordance with these recommendations [23]*.* Another retrospective single-center study from 2014 to 2017 reported that 47% of LVO strokes in the anterior circulation were eligible for EVT according to AHA/ASA guidelines [24]. In our study, nearly half of patients who received EVT in real world did not meet RCT criteria, suggesting remaining ambiguities in patient selection, also because observed outcomes at 90 days after EVT were worse outside the RCT criteria than within the RCT criteria. These findings highlight a critical gap between clinical trial conditions and everyday clinical practice. Our results advocate for future studies to explore and validate more inclusive criteria and to identify which subsets of patients currently excluded from RCTs might still benefit from EVT. This would help in developing more comprehensive and applicable guidelines, ultimately improving patient selection and outcomes for EVT.

To investigate off-label EVT, we first analyzed the main exclusion criteria and their association with functional outcome. Our data showed that the most common exclusion reason for potential trial participation was a prestroke functional dependence in 62%, which logically prevents the achievement of functional independence during the course. A large infarct core respectively low ASPECTS as well as unknown or a largely extended time window showed no independent association in our cohort, however, were put into perspective by more recent trials [25–27]. The other exclusion criteria that showed an association with functional outcome, namely a too-low NIHSS and a peripheral vascular occlusion, were per se associated with a favorable outcome, making a risk-benefit consideration even more prominent here. Overall, favorable functional outcome after off-label treatment was still achieved in 22% which argues against absolute adherence to the aforementioned exclusion criteria in real-world.

Second, we investigated association with functional outcome among off-label treated patients. The most frequent reason for off-label EVT was a prior disability, but this was not independently associated with the composite endpoint of regaining functional independence or prestroke functional status. However, our multivariable analysis showed an independent association of functional recovery with lower NIHSS and younger age. Therefore, we can hypothesize that previous disability did not prevent patients from recovering to pre-stroke functional status, but older age and clinically severe stroke did. To illustrate, the probability of an impaired patient with no loss of ambulation without assistance from another person (mRS score 3) regaining or maintaining this functional status after off-label EVT, as opposed to loss of ambulation without assistance from another person or loss of the ability to perform bodily self-care (mRS score 4), the need for continuous care (mRS score 5) or a fatal outcome (mRS score 6), appears to be more related to NIHSS and age than to the pre-existing impairment. We also documented univariate associations with fewer comorbidities and less frequent anticoagulation, higher ASPECT score, or smaller infarct core, i.e., overall pathophysiologically consistent associations that may not have been significant in multivariate analysis because of limited statistical power in this subgroup.

Third, we assessed treatment effects of off-label EVT, showing only an effect on mortality but not on functional outcome in our real-world cohort. This finding highlights the importance of improved patient selection to identify those who actually benefit from EVT. Therefore, individualized decision making should continue for each patient outside the trial inclusion criteria, based on the known clinical and radiological outcome predictors after ischemic stroke, with particular consideration of stroke severity on admission and patient age [28–30]. Given the observed limited effect of off-label EVT on functional outcome, patient selection should always include ethical decision-making respecting the patient`s will regarding potential survival with major functional disability. For instance, recent studies have demonstrated the potential of EVT to benefit patients with large infarct cores, yet even in these intervention groups, a significant proportion of patients remain severely disabled [31].

This study has several limitations. First, the generalizability of these results may be limited by the EVT data originating solely from the coordinating center of a large telemedicine network and its retrospective analysis. Second, the sample size and thus the statistical power was small in the cohort with EVT in the extended time window, so an undetected difference in the achieved outcomes between patients from the RCTs compared to the real world cannot be excluded in these cases. Third, although we applied firm statistical means such as propensity score matching, we cannot exclude residual unmeasured confounding especially in control patients not treated with EVT. Fourth, outcome assessment consisted only of the mRS and data on patient-centered outcomes or health-related quality of life were not available. These measurements have also been shown to be positively influenced by successful EVT, and represents another important dimension of stroke outcomes for patients in addition to purely functional measurements [32]. Future studies should include these measures to provide a more comprehensive understanding of the impact of EVT outside of RCT criteria on patients' quality of life.

## Conclusions

In conclusion, this study documented that the favorable outcomes after EVT reported in RCTs can also be achieved in routine clinical practice. However, many patients are treated outside the trial criteria, and this study hints towards a reduced mortality with only limited effect on functional outcome after EVT. Therefore, further randomized trials with broader inclusion criteria seem warranted to improve patient selection for EVT.

### Supplementary Information

Supplementary Material 1. 

## Data Availability

The data that support the findings of this study will be made available upon reasonable request and in adherence with institutional data sharing regulations. Requests should be directed to the corresponding author.

## Abbreviations

EVT: Endovascular thrombectomy
CI: Confidence Interval
LVO: Large vessel occlusion
mRS: modified Rankin scale
NIHSS: National Institutes of Health Stroke Scale
OR: Odds ratio
RCT: Randomized controlled trial
