# Supplementary material for: Endovascular thrombectomy in patients with anterior circulation stroke: an emulated real-world comparison
Source: Neurol Res Pract. 2024 Jul 25;6:37. doi: 10.1186/s42466-024-00331-6 (PMC11270839; doi:10.1186/s42466-024-00331-6)
Supplement: Supplementary file 1 — Supplementary Material 1. [file 42466_2024_331_MOESM1_ESM.docx]

**Supplementary material**

*Sembill et al.*

Endovascular thrombectomy in patients with anterior circulation stroke:

An emulated real-world comparison

Content:

**Supplemental Table 1.** Main clinical trial participation criteria

**Supplemental Table 2**. Characteristics of patients included vs. excluded into the STAMINA-HERMES or STAMINA-AURORA emulation cohort

**Supplemental Figure 1.** Functional outcome of patients included either into STAMINA-HERMES or into STAMINA-AURORA cohort compared to patients of the STAMINA-OFF-LABEL cohort

**Supplemental Table 3.** Characteristics of patients from the STAMINA-OFF-LABEL cohort with vs. without recovery to functional independence or premorbid status

**Supplemental Table 4.** Characteristics of patients from the STAMINA-OFF-LABEL cohort compared to controls with anterior circulation stroke without endovascular thrombectomy

**Supplemental Table 1.** Main clinical trial participation criteria

| Trial name | MR CLEAN | ESCAPE | REVASCAT | SWIFT PRIME | EXTEND IA | DAWN | DEFUSE 3 | RESILIENT | POSITIVE |
| --- | --- | --- | --- | --- | --- | --- | --- | --- | --- |
| Age, years | ≥18 | ≥18 | 18-80 (later amended to 81-85 if ASPECTS>8) | 18-85 (later amended to 18-80) | ≥18 | ≥18 | ≥18–90 | ≥18 | ≥18 |
| Pre-morbid status | N/A | Barthel ≥90 | mRS 0-1 | mRS 0-1 | mRS 0-1 | mRS 0-1 | mRS 0-2 | mRS 0-1 | mRS 0-1 |
| Stroke severity | NIHSS ≥2 | NIHSS ≥6 | NIHSS ≥6 | NIHSS ≥8 and <30 | N/A | NIHSS ≥10 | NIHSS ≥6 | NIHSS ≥8 | NIHSS ≥8 |
| Time to randomization | 6 hours | 12 hours | 8 hours | 6 hours | 6 hours | 6–24 hours | 6–16 hours | 6–8/12 hours | 6–12 hours |
| Vessel occlusion | ICA, M1, M2, A1, A2 | ICA, M1 | ICA, M1 | ICA, M1 | ICA, M1, M2 | ICA, M1 | ICA, M1 | ICA, M1 | ICA, M1 |
| Minimum requirement for baseline infarct / perfusion imaging | N/A | ASPECTS 6-10, Good collaterals | ASPECTS-CT 7-10 or ASPECTS-MRI 6-10 | ASPECTS 6-10 | Mismatch >10ml or ratio >1.2, ischemic core <70mL | Infarct volume  <21ml if ≥80y,  <31ml if <80y,  31 to <51 mL if <80y and NIHSS ≥20 | ASPECTS 6-10, Infarct volume <70 ml, mismatch ratio  ≥1.8 and penumbra  of ≥15 ml | ASPECTS 6-10, Infarct volume <70 ml, mismatch ratio  ≥1.8 and penumbra  of ≥15 ml | ASPECTS 7-10, significant penumbra according to each center’s institutional protocol |

The NIHSS (range 0 to 42) indicates stroke severity, with higher scores indicating greater severity). The premorbid mRS ranges from 0 (no symptoms) to 5 (severe disability). Abbreviations: ASPECTS, Alberta Stroke Program Early CT Score; ICA, Internal Carotid Artery; MCA, Middle Cerebral Artery; NIHSS, National Institutes of Health Stroke Scale; mRS, modified Rankin Scale.

**Supplemental Table 2**. Characteristics of patients included vs. excluded into the STAMINA- HERMES or STAMINA-AURORA emulation cohort

| **Parameters** | **STAMINA-HERMES or STAMINA- AURORA emulation cohort (n=321)** | **STAMINA-OFF-LABEL cohort (n=277)** | **p-value** |
| --- | --- | --- | --- |
| Demographics |  |  |  |
| Age, median (IQR), years | 73 (59-79) | 78 (67–84) | <0.001 |
| Female Sex, No. (%) | 166 (52) | 169 (61) | 0.02 |
| Prior medical history |  |  |  |
| Hypertension, No. (%) | 246 (77) | 228 (82) | 0.09 |
| Diabetes mellitus, No. (%) | 85 (26) | 89 (32) | 0.13 |
| Atrial fibrillation, No. (%) | 144 (45) | 129 (47) | 0.67 |
| Smoking (recent or current), No. (%) | 80 (25) | 64 (23) | 0.60 |
| Hyperlipidaemia, No. (%) | 156 (49) | 132 (48) | 0.82 |
| Oral anticoagulation, No. (%) | 59 (18) | 52 (19) | 0.89 |
| Pre-mRS, median (IQR) | 0 (0-1) | 2 (0-3) | <0.001 |
| Clinical characteristics |  |  |  |
| Baseline NIHSS Score ^a^, median (IQR) | 15 (11-19) | 16 (11–20) | 0.28 |
| Imaging characteristics |  |  |  |
| Baseline ASPECT Score ^b^, median (IQR) | 9 (7-10) | 9 (7–10) | 0.61 |
| Ischemic core lesion volume, median (IQR), mL | 5 (0-22) | 5 (0-34) | 0.35 |
| Mismatch volume, median (IQR), mL | 105 (71-153) | 94 (40-140) | 0.02 |
| Intracranial occlusion location |  |  |  |
| Internal carotid artery, No. (%) | 112 (35) | 89 (32) | 0.48 |
| Middle cerebral artery, M1 segment, No. (%) | 167 (52) | 127 (46) | 0.13 |
| Middle cerebral artery, M2 segment, No. (%) | 42 (13) | 57 (21) | 0.01 |
| Middle cerebral artery, M3 segment, No. (%) | 0 (0) | 3 (1) | 0.10 |
| Anterior cerebral artery, No. (%) | 1 (0) | 1 (0) | 1.00 |
| Treatment |  |  |  |
| Treatment with intravenous alteplase, No. (%) | 245 (76) | 161 (58) | <0.001 |
| Treatment with intravenous alteplase  within 180 min, No. (%) | 157 (49) | 70 (25) | <0.001 |
| TICI scale ^c^ ≥2b, No. (%) | 296 (92) | 230 (83) | <0.001 |
| Intracerebral hemorrhage, No. (%) | 28 (9) | 27 (10) | 0.66 |
| Process times |  |  |  |
| Onset to i. v. alteplase, median (IQR) min | 95 (75-130) | 115 (90–173) | 0.002 |
| Onset to reperfusion, median (IQR) min | 325 (238-464) | 465 (314–924) | <0.001 |

^a^ NIH Stroke Scale, range 0-42, level of neurological impairment after stroke. ^b^ Alberta Stroke Program Early CT Score, range 0-10, assessment of early ischemic changes in acute stroke ^c^ Thrombolysis in cerebral infarction (TICI) Score, range 0-3, from no perfusion to complete perfusion. Abbreviations: IQR, Interquartile range (25th-75th percentile).

**Supplemental Figure 1.**

Functional outcome of patients included either into STAMINA-HERMES or into STAMINA-AURORA cohort compared to patients of the STAMINA-OFF-LABEL cohort


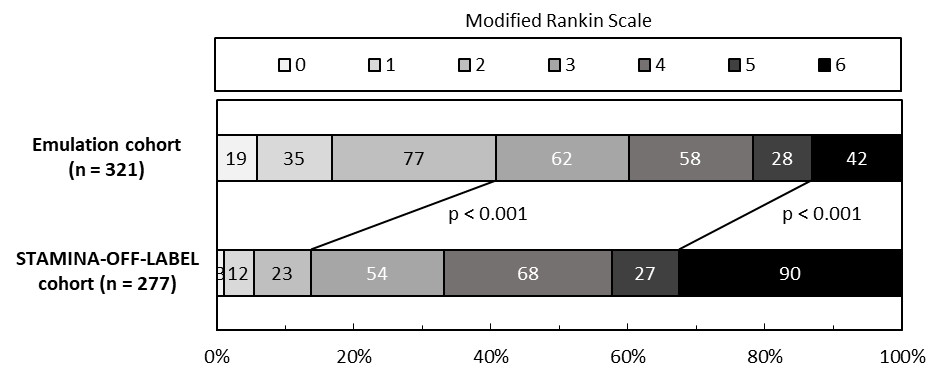


Shown is the distribution of scores on the modified Rankin scale at 90 days in patients treated in-line with trial inclusion criteria, i.e. the emulation cohort, vs. outside trial inclusion criteria, i.e. the STAMINA-OFF-LABEL cohort. The Scores range from 0 to 6, with 0 indicating no symptoms, 1 no clinically significant disability, 2 slight disability, 3 moderate disability, 4 moderately severe disability, 5 severe disability, and 6 death. Scores from 0 to 2 are considered functional independence.

**Supplemental Table 3.**

Characteristics of patients from the STAMINA-OFF-LABEL cohort with vs. without recovery to functional independence or premorbid status

| **Parameters** | **No functional recovery (n=215)** | **Functional recovery (n=62)** | **p-value** |
| --- | --- | --- | --- |
| Demographics |  |  |  |
| Age, median (IQR), years | 80 (72–85) | 69 (55-79) | <0.001 |
| Female Sex, No. (%) | 138 (64) | 31 (50) | 0.04 |
| Prior medical history |  |  |  |
| Hypertension, No. (%) | 184 (86) | 44 (71) | 0.01 |
| Diabetes mellitus, No. (%) | 75 (35) | 14 (23) | 0.07 |
| Atrial fibrillation, No. (%) | 109 (51) | 20 (32) | 0.01 |
| Smoking (recent or current), No. (%) | 45 (21) | 19 (31) | 0.11 |
| Oral anticoagulation, No. (%) | 46 (21) | 6 (10) | 0.04 |
| Pre-mRS, median (IQR) | 2 (1-3) | 1 (0-3) | 0.14 |
| Clinical characteristics |  |  |  |
| Baseline NIHSS Score ^a^, median (IQR) | 17 (13-21) | 10 (5–16) | <0.001 |
| Imaging characteristics |  |  |  |
| Baseline ASPECT Score ^b^, median (IQR) | 9 (7-10) | 10 (8-10) | 0.04 |
| Ischemic core lesion volume, median (IQR), mL | 8 (0-38) | 0 (0-19) | 0.04 |
| Mismatch volume, median (IQR), mL | 100 (47-143) | 72 (38-125) | 0.08 |
| Intracranial occlusion location |  |  |  |
| Internal carotid artery, No. (%) | 72 (34) | 17 (27) | 0.37 |
| Middle cerebral artery, M1 segment, No. (%) | 99 (46) | 28 (45) | 0.90 |
| Middle cerebral artery, M2 segment, No. (%) | 40 (19) | 17 (27) | 0.13 |
| Middle cerebral artery, M3 segment, No. (%) | 3 (1) | 0 (0) | 0.59 |
| Anterior cerebral artery, No. (%) | 1 (1) | 0 (0) | 1.00 |
| Treatment |  |  |  |
| Treatment with intravenous alteplase, No. (%) | 126 (59) | 35 (57) | 0.76 |
| Treatment with intravenous alteplase  within 180 min, No. (%) | 56 (26) | 14 (23) | 0.58 |
| TICI scale ^c^ ≥2b, No. (%) | 176 (82) | 54 (87) | 0.33 |
| Intracerebral hemorrhage, No. (%) | 25 (12) | 2 (3) | 0.05 |
| Process times |  |  |  |
| Onset unknwon, No. (%) | 118 (55) | 32 (52) | 0.65 |
| Onset to i.v. alteplase, median (IQR) min | 120 (90-180) | 98 (75–167) | 0.35 |
| Onset to reperfusion, median (IQR), min | 458 (299-946) | 467 (344–853) | 0.99 |

^a^ NIH Stroke Scale, range 0-42, level of neurological impairment after stroke. ^b^ Alberta Stroke Program Early CT Score, range 0-10, assessment of early ischemic changes in acute stroke ^c^ Thrombolysis in cerebral infarction (TICI) Score, range 0-3, from no perfusion to complete perfusion. Abbreviations: IQR, Interquartile range (25th-75th percentile)

**Supplemental Table 4**.

Characteristics of patients from the STAMINA-OFF-LABEL cohort compared to controls with anterior circulation stroke without endovascular thrombectomy

| **A) Before propensity score matching** |  |  |  |
| --- | --- | --- | --- |
| **Parameters** | **STAMINA-OFF-LABEL cohort (n=277)** | **Non-EVT controls (n=200)** | ***p*-value** |
| Demographics |  |  |  |
| Age, median (IQR), years | 78 (67–84) | 81 (71-87) | 0.001 |
| Female Sex, No. (%) | 169 (61) | 124 (62) | 0.83 |
| Prior medical history |  |  |  |
| Hypertension, No. (%) | 228 (82) | 156 (78) | 0.24 |
| Diabetes mellitus, No. (%) | 89 (32) | 55 (28) | 0.28 |
| Atrial fibrillation, No. (%) | 129 (47) | 106 (53) | 0.17 |
| Smoking (recent or current), No. (%) | 64 (23) | 29 (15) | 0.02 |
| Hyperlipidaemia, No. (%) | 132 (48) | 90 (45) | 0.57 |
| Oral anticoagulation, No. (%) | 52 (19) | 30 (15) | 0.28 |
| Pre-mRS, median (IQR) | 2 (0-3) | 3 (2-3) | <0.001 |
| Clinical characteristics |  |  |  |
| Baseline NIHSS Score ^a^, median (IQR) | 16 (11–20) | 15 (8-20) | 0.44 |
| Imaging characteristics |  |  |  |
| Baseline ASPECT Score ^b^, median (IQR) | 9 (7–10) | 8 (6-10) | <0.01 |
| Ischemic core lesion volume, median (IQR), mL | 5 (0-34) | 19 (0-75) | <0.001 |
| Mismatch volume, median (IQR), mL | 94 (40-140) | 55 (24-95) | <0.001 |
| Intracranial occlusion location |  |  |  |
| Internal carotid artery, No. (%) | 89 (32) | 73 (37) | 0.32 |
| Middle cerebral artery, M1 segment, No. (%) | 127 (46) | 73 (37) | 0.04 |
| Middle cerebral artery, M2 segment, No. (%) | 57 (21) | 49 (25) | 0.31 |
| Middle cerebral artery, M3 segment, No. (%) | 3 (1) | 3 (2) | 1.00 |
| Anterior cerebral artery, No. (%) | 1 (0) | 2 (1) | 0.57 |
| Treatment |  |  |  |
| Treatment with intravenous alteplase, No. (%) | 161 (58) | 92 (46) | <0.01 |
| Treatment with intravenous alteplase  within 180 min, No. (%) | 70 (25) | 45 (23) | 0.48 |
| **B) After propensity score matching** |  |  |  |
| **Parameters** | **STAMINA-OFF-LABEL cohort (n=128)** | **Matched Non-EVT controls**  **(n=128)** | ***p*-value** |
|  |  |  |  |
| Age, median (IQR), years | 80 (70–85) | 80 (70-87) | 0.48 |
| Pre-mRS, median (IQR) | 3 (1-3) | 3 (0-3) | 0.44 |
| Baseline ASPECT Score ^b^, median (IQR) | 8 (7–10) | 9 (7-10) | 0.57 |
| Ischemic core lesion volume, median (IQR), mL | 5 (0-35) | 10 (0-48) | 0.27 |
| Mismatch volume, median (IQR), mL | 75 (38-117) | 60 (28-119) | 0.27 |
| Treatment with intravenous alteplase, No. (%) | 74 (58) | 83 (65) | 0.25 |

Comparison of patients from the STAMINA-OFF-LABEL cohort, i.e. patients with anterior ischemic stroke due to large vessel occlusion treated with endovascular thrombectomy (EVT) outside clinical trial criteria, compared to Non-EVT controls, i.e. patients with anterior ischemic stroke due to large vessel occlusion not treated with EVT. Shown are patient characteristics A) before propensity score matching and B) balanced parameter with prior differences between cohorts after propensity score matching.

^a^ NIH Stroke Scale, range 0-42, level of neurological impairment after stroke.

^b^ Alberta Stroke Program Early CT Score, range 0-10, assessment of early ischemic changes in acute stroke

^c^ Thrombolysis in cerebral infarction (TICI) Score, range 0-3, from no perfusion to complete perfusion

Abbreviations: IQR, Interquartile range (25th-75th percentile)
